# Annotations

**Published:** 1911-06-10

**Authors:** 


### ANNOTATIONS.

### The N.S.P.C.C.

There are many satisfactory features about the Annual Report of the National Society for the Prevention of Cruelty to Children, a copy of which has been forwarded to this office. It is unfortunately true that the number of cases reported to the Society for inquiry is the largest on record, as is also the number found upon investigation to need the interference of the Society. But though that is so, there is so large an increase in the number of cases in which warnings are sufficient that the cases demanding more stringent measures are considerably fewer than last year. Two reasons are given for this satisfactory state of affairs: one that the power of the law is more appreciated by offenders, and the fear of prosecution is greater; and the other that an increased staff of inspectors has resulted in closer attention to individual cases. Another important point is that the percentage of cases reported by the Society's inspectors shows a diminution, thus disposing of the charge that these officials tend to manufacture cases: in fact, only 8.8 per cent. of the Society's cases are thus discovered, the general public itself reporting more than half the total cases on the list. Prosecutions in court have diminished notably, and have been fewer than for fifteen years; about 97 per cent. of these prosecutions are successful. There are many more figures in this report which show in how many ways the work is well done and worth doing; always is prevention kept in view and preferred to the punishment of offences committed. Now and then the publication of the details of some shocking police-court trial for cruelty outrages public feeling and draws attention in a dramatic way to the Society's beneficent activity. It should be realised that that activity goes on unceasingly and that for every such glaring and sensational case there are many thousands dealt with in which protection is just as much required for some pitiable child-victim.

### The Yellow Fever Bureau Bulletin.

The first number of a periodical bearing the above title has reached us by the courtesy of the editorial committee and the Liverpool School of Tropical Medicine, under whose auspices the *Bulletin* appears. The promoter of the enterprise seems to be Sir Rubert Boyce, already well known for his writings on this and other diseases of tropical climates; at least this inference is suggested by the facts that Sir Rubert's name appears on the inside cover as the receiver of all communications in respect of the Yellow Fever Bureau, and that a considerable part of the first issue is devoted to papers by this author. Sir R. Boyce upholds a theory that yellow fever is much more widespread and much more prevalent in certain parts of the tropics than is usually admitted or believed; that it is frequently overlooked or misdiagnosed; and that even when it is discovered it is often not reported because of the reluctance of administrations and civil populations to become known as endemic centres of this disease. If this theory is entirely true, or is even partly true, it is evident that all sorts of momentous consequences flow from it; and that the sanitation of tropical colonies, and especially of tropical towns, must include systematic extermination of the *Stegomyia fasciata*. The difficulty in determining whether the numerous cases reported as bilious remittent fever, inflammatory fever, malaria with gastritis, gastric fever, etc., are really mild or unsuspected cases of yellow fever is enhanced by the lack of any certain means of diagnosing the latter microscopically. The parasite of yellow fever is still unidentified, and hæmolytic tests are, of course, out of the question. The diagnosis rests, therefore, purely on clinical signs. There can be no doubt that the publication of a monthly periodical, especially devoted to this one disease, is bound to result in an increase both of the interest taken in it and of the knowledge we have of it. The *Bulletin* is excellently produced, and can hardly fail to serve a most useful purpose.

### Recent Literature by Medical Authors.

Two volumes of reminiscences have lately appeared which should prove of interest to the medical profession. The first is Dr. Farquharson's vivacious record of parliamentary life, entitled "In and Out of Parliament." In it we have pictures of many of the most memorable events in the political history of the last thirty years, during which, we are bound to repeat, the influence of medical men in the House has been sadly, if inevitably, wanting. For twenty-six years, Dr. Farquharson was able to look upon many famous scenes and study the characters of the prominent men of his time, and this illuminating commentary is one well worth perusing. The other book deals with the recollections of a Parisian of the first half of the last century, Dr. Poumiès de la Siboutie. Living as he did under six sovereigns, three revolutions, and two republics, he had need to be a philosopher; indeed, he appears to have had a good store of the gifts of human sympathy and observation. Dr. de la Siboutie has so many charming anecdotes to tell and so many interesting sidelights are shed on his great contemporaries that the book is undoubtedly one of the most entertaining volumes of reminiscences of recent years.

### The Infanticide Bill.

At last steps are being taken to amend the law relating to infanticide, and the death sentence passed when there is no intention whatever of carrying it out, will, it is hoped, be abolished before long. In the present state of the law, when any woman has been convicted of infanticide, the judge has no alternative but to pass sentence of death, and a scene distressing alike to prisoner and judge has to be gone through. Mr. Palmer's Bill provides that in certain cases the judges shall be empowered to pass the same sentence as if the prisoner had been convicted of manslaughter only.

